# The New York Institute of Stomatology

**Published:** 1902-02

**Authors:** 

**Affiliations:** The New York Institute of Stomatology


### Reports of Society Meetings.

### THE NEW YORK INSTITUTE OF STOMATOLOGY.

A regular meeting of the New York Institute of Stomatology was held on Tuesday evening, October 1, 1901, at the residence of Dr. MacNaughton, No. 63 West Forty-ninth Street, New York City.

The President, Dr. J. Morgan Howe, occupied the chair and called the meeting to order.

The Secretary read the minutes of the last regular meeting, which were approved.

### SPECIAL BUSINESS.

*The President.*—Section 9, Article II., of the By-laws, provides that at the October meeting the President shall appoint two members, who, with the Executive Committee, shall constitute a Committee of Nomination for all the officers to be elected at the annual meeting. In accordance with that requirement, I will appoint Drs. C. B. Parker and J. B. Locherty, who, with the Executive Committee, will constitute a Nominating Committee for the ensuing year.

I have a note from Dr. Bogue, to inform us that our fellowmembers, Dr. Cowardin, of London, and Dr. Bellaserio, of Sydney, Australia, have recently died. Can Dr. Bogue give us any information further than that contained in his letter?

*Dr. E. A. Bogue.*—I cannot, excepting that Dr. Bellaserio, as I suppose is generally known, was one of Sir John Tomes's old friends, and was full of years at the time of his death, which was expected. Dr. Cowardin, a young man, was here with us a few months ago, and died very suddenly. Dr. Cowardin's brother lives in Baltimore.

*Dr. S. E. Davenport.*—It is a painful duty I perform in officially announcing to the Institute the death, June 16, 1901, of Zachary T. Sailer, D.D.S. Dr. Sailer was one of the twenty-three charter members of this organization, and is the third which the Institute loses of that number by death, the other two being Dr. Charles F. Ives and Dr. Charles W. Meloney.

Dr. Sailer was the Institute's first curator, occupying that office until the latter part of 1897, when on account of ill health he resigned from the list of officers, retaining, however, his active membership.

Those of us who came often in contact with Dr. Sailer either professionally, socially, or in the transaction of the business of the Institute, invariably found him genial and courteous, resourceful in the management of affairs, and a gentleman with whom it was always pleasant to associate.

During the last two years Dr. Sailer's health prevented his meeting with us, but that he was interested in the work of the society was shown by several letters he wrote lamenting his inability to attend meetings as formerly.

The universal feeling in our membership is, I am sure, one of sorrow and deep regret for the loss which the Institute has sustained in the death of Dr. Sailer.

*The President.*—I am sure we all sympathize with the expression just made with regard to our loss in Dr. Sailer's death, and I do not know that it is necessary that a motion should be made to receive the communication and make it a part of our proceedings of the evening. I think it would be so understood.

### COMMUNICATIONS ON THEORY AND PRACTICE.

*Dr. C. O. Kimball.*—It came to the knowledge of the Executive Committee very late this summer that at the meeting of the

National Dental Association last year it was voted to receive as delegates members from all recognized dental societies who accepted the ethical standard of the National Dental Association as their standard. By some mistake, the notice was not sent to us in time to bring the matter formally before the entire body of members, and, acting for the Board of Directors, the Executive Committee authorized the President—or rather the Vice-President acting in the absence of the President—and the Secretary to appoint two members to represent the Institute at the National Dental Association at Milwaukee. The meeting was an interesting one, and calculated to do much good. Dr. Bogue and myself, who were there, may possibly later on have something to say about the details of the gathering. I wish to report formally our acceptance of the invitation.

*The President.*—I am sure we are all gratified that such action was taken. Of course we wish to perform our duties as related to the national body, and we are much obliged to the members who acted as delegates. If Dr. Bogue has anything to tell us about the meeting that he thinks would be interesting, it would be acceptable at this time.

*Dr. E. A. Bogue.*—We have heretofore not been represented in the National Dental Association, and there was a very considerable effort made that none but State societies should be represented in that body. One of our members, Dr. Wheeler, made a good deal of effort at Old Point Comfort to promote the resolution that was finally passed, admitting to membership delegates from all reputable and well-organized dental bodies that had been in existence a year and recognized the code of ethics. As Dr. Kimball and myself will remain permanent members, I presume, it will be in order for the Institute to remember that there will be vacancies for the ensuing year, and I hope that better delegates, or those better qualified, will go next year to represent this body and work for it, and that we may come into closer relationship with an institution which is calculated to do, as Dr. Kimball has just said, great good, and which has it in its power very largely to influence our calling.

It may be known to many present that a number of bogus diplomas have been issued to men on the other side of the water, and some on this side. That matter was taken up at the meeting, and with the assistance of the American Consul at Munich, Mr. Worman, efforts are now being made to have the law of Illinois so construed that improper licensing or diploma-ing "*in absentia*" cannot be made. Governor Yates, of Illinois, has been interested, and something like three thousand six hundred dollars (I speak from memory only) was voted by the two bodies—the National Dental Association and the National Association of Dental Faculties—to carry out this plan, with Consul Worman as its mouthpiece and Dr. Crouse as its actuary.

*Dr. George S. Allan.*—My head is so full of yachts, and blue skies, and salt water, that I had almost forgotten this communication which I wish to present. I brought this with me, not because I think there is anything particularly new about it,—there is nothing new under the sun, anyway, especially in mechanical dentistry,—but it contains a principle that I have used in my practice considerably, and with very beneficial results, so I had my plate-worker, while making the one that is to be set to-morrow, make a duplicate to show the Institute. The patient, who is about forty years old or a little more, had practically lost the use of the left side of the face, although she has the full number of teeth in the upper jaw, and has lost only one in the lower jaw; but the two molars, as you will see when this cast is passed around, are so badly decayed on their approximal faces and grinding surfaces that the food crowds in against the gum in a most annoying and distressing way, and keeps the gum irritated so that she cannot eat on that side with any comfort. The twelfth-year molar is a living tooth; the wisdom-tooth is dead and very badly decayed indeed. It would have been possible to put in a very large filling on the grinding surface of the twelfth-year molar, but it would have been nearly impossible to fill the wisdom-tooth in the same way, to have the spaces occlusal and prevent the crowding in of food. So I made this little appliance, which you will see fits over the two teeth and is to be cemented in place. In brief, it is two rings, bands, one on each molar, soldered together on their approximal surfaces. If the patient were going to remain in this country, I would have had her use this appliance without cementing, and the probability is it would have been equally successful, and could have been kept clean, as I have made similar appliances, and had them work very satisfactorily. There is no rapid decay going on in the mouth, and by watching such an appliance as this, there is no real necessity why there should be any large amount of decay. The patient could remove it, clean her teeth, and put it back; but as she is to be gone a year in Europe, I have decided to cement it in place. The appliance explains itself. The gums will be perfectly protected when it is in place. The toothpick goes underneath and keeps the space clear, and as the articulation is very good, I feel certain that I will restore to this patient full use of both sides of her mouth. As I say, there is nothing really new about it, but possibly it may be new to some of you,—the way in which the thing has been shaped.

While the cross-piece goes between the bicuspid and the twelfth-year molar, it simply goes between the teeth without going down to the gingival border, so the floss-silk can be used.

*Dr. F. Milton Smith.*—I have two or three little things here that I thought might be of interest. I first call attention to a lateral incisor tooth, that two or three days ago my patient broke off at the gum line. It is interesting to me for many reasons. The principal one is the very large contour filling (proportionately to the size of the lateral incisor tooth), which was put in nearly six years ago, and which is anchored at the lower end or cutting edge with oxyphosphate of zinc cement. The tooth at that time was very frail, and had been filled and refilled,—at one time the patient's teeth decayed very rapidly,—and when the contour filling which had been in previously to this failed, the cutting edge of the tooth where we ordinarily get the anchorage was so thin that I felt it was absolutely impossible for me to depend on that edge if I malleted gold in the undercut. I will pass the tooth around in a moment. What is left of it is almost entirely enamel on the outside. The cement was put in in its plastic condition, as many men are accustomed to doing nowadays, and, as I remember it, White's mat gold was embedded in the soft material. After the phosphate had hardened, the gold was thoroughly condensed with hand-instruments. The upper portion was retained by undercuts in and about the neck of the tooth. The labial filling had been in some years previous to that. What I want to call attention to is the fact that it stood for six years with that kind of anchorage, and I do not believe it would have been possible, if I had malleted gold in it, to have had it stand until I finished the operation. So little of the tooth-substance remaining, the patient having fillings almost as extensive of gold as this one, I did not want to put in a porcelain tooth, because it would have been unsightly in connection with the other teeth. I found, greatly to my surprise, that the pulp was alive. The patient was forty-five years of age. In order that it might be restored as nearly as possible as this tooth is, I had prepared a gold-band crown to which I added a gold face, and then cut out sufficient of the gold face for my porcelain, as I have it here. I purpose to make a porcelain inlay and cement it in this place, and think when I shall have set it I shall succeed in deceiving the patient's friends so they will not know she has lost a tooth.

Another thing is of interest to me in the porcelain inlay line. I presume some of the gentlemen present have had difficulty in getting disks sufficiently thin and fine to cut the retaining grooves around small inlays. Dr. Allan had in his instrument-case a comparatively fine disk, which had been the best he was able to secure. I borrowed it from him, showed it to White's man, and he claimed it was the best made, and said he would send me one like it. I found that for the purpose of cutting small inlays it was absolutely of no account to me. Almost immediately on getting it, a copy of the *Dental Brief* came to me, and in the items of interest (I do not know under what head they are classed there) some one made the suggestion, which, of course, is not new to any of us, that copper charged with carborundum powder or diamond dust would cut the porcelain, and suggested that a very thin piece of copper might be soldered on to a mandrel and charged with carborundum powder and glycerin. I accepted the suggestion, and tried these little disks. I meant to bring a tooth in which I had made a large number of cuts, so you might see how readily they cut. It is very simple to make them, if any one is mechanically inclined, and almost every man is,—certainly every one connected with this Institute,—and if you do not want to make them, your instrument-maker will do it for you. The White's disk is also in the box, for comparison.

*The President.*—If there is nothing further under this head, we will pass to the important communication of the evening, which is to be furnished us by Dr. Samuel A. Hopkins, of Boston, who will read a paper on "Science as a Teacher of Prophylaxis."

(For Dr. Hopkins's paper, see page 80.)

DISCUSSION.

*The President.*—Gentlemen, this very interesting and suggestive paper of Dr. Hopkins is before you for discussion. We invite all present to take part in discussing it.

*Dr. S. E. Davenport.*—The more we learn of science, the more do we learn that it is but the basis of practical, common-sense reasoning. There was a time when we were afraid if the word "science" was used; it seemed beyond us; but Dr. Hopkins has a way of expressing the truths of science in understandable language, for which I am personally obliged. We are unfortunate in one thing, however, Dr. Hopkins belongs to many societies, and the work of his hands and brain has to be distributed. We have lost the first chapter of a very interesting story, and while Dr. Hopkins was kind enough to quote a few headings from it, we are left in the dark regarding the carrying out of those thoughts. The first paper on this subject, as I understand, was read at the June meeting of the Massachusetts Dental Society, and I have been informed to-night that a continuation of this subject is to be given by Dr. Hopkins before the Northeastern Dental Society, which meets the last of October. I am very glad the movement is on foot for a number of our members to go to that meeting at Springfield.

One of the points of Dr. Hopkins's first paper related, he tells us, to the difference between living teeth and pulpless teeth, in their ability to resist decaying influences, and I shall read his paper as soon as I have the opportunity, as much to learn his position upon that very important question as for any other one thing. I am hoping he will have taken the position that the dental pulp is of enormous value in the adult tooth. There is a large school of dentists at the present time who not only practise but preach that the dental pulp is but a formative organ,—one simply of development,—and that it has no particular value in the adult tooth. I believe that to be a mistaken idea, and anything which is said based upon scientific investigation which shall incline us to the preservation in all possible cases of the dental pulp will, I believe, be for the good of our patients.

A very important point which Dr. Hopkins referred to in his paper this evening is that of the necessity for the proper occlusion of teeth, in order that the best conditions may be established and continued. That is a point to which too little attention is given. Extractions are advised, many times carelessly and without due study of the casts as well as of the mouth, with the mistaken idea that the correction of the deformity will be made easier and that the teeth will rearrange themselves after the extraction. The occlusion of the remaining teeth in that mouth is often so interfered with that not only are the teeth more difficult to cleanse because of their irregularity and abnormal position, but elongations of certain teeth occur, and a general lack of symmetry results.

*Dr. E. H. Babcock.*—There is one point I would like to speak about, not so much to express any fact myself, as to get the views of the other members of the society. Dr. Hopkins spoke of the great rapidity in the process of decay in mouths where the saliva was thick and ropy. I have several cases—two brothers in particular—whose teeth decay very rapidly, and the saliva has always been very ropy, but I notice it varies at different times. As Dr. Hopkins spoke of the matter, the question presented itself whether the increased decay was due to the decomposition of the organic material which is undoubtedly in that saliva, making it ropy, or whether that organic material merely acted as a pabulum for the micro-organisms.

There was one other question about the cleansing of the teeth. Dr. Hopkins referred to Dr. D. D. Smith, of Philadelphia. I was present at a meeting where Dr. Smith discussed the question, and I was very glad he brought out the points as forcibly as he did; but one thing I think he rather overdid, and that is, he spurned the dental engine altogether. I think the stick on which he lays such stress is all right; but I think you can supplement it with the engine, with less waste of time and with more pleasure to the patient. I would like to have Dr. Hopkins's view of the ropy saliva, if he can spare the time.

*Dr. Kimball.*—I came down from Lake George last night purposely to hear Dr. Hopkins's paper, and expect to go back to-night. I did so, I am free to confess, very largely as a matter of courtesy to Dr. Hopkins, as he is an old and dear friend of mine. I had supposed I would be through with my summer vacation when this paper was read, and it required quite a struggle for me to leave last night. I will say that the paper has far exceeded what I looked for in the way of a full statement of the scientific principles involved, and of the necessary and proper deductions from them, and I cannot help but think how much good will be done by the spreading abroad of the report of this paper when published. Turning our thoughts in that direction, we owe much to a good many men, among them Dr. D. D. Smith, who honors us with his presence this evening. Looking back, we see that the leaders in professional life have been the men who have paid attention to this very point,—care of teeth from a hygienic point of view,—who have endeavored so far as they knew to resist decay, and I am sure in the future the outcome of such papers as this and the thoughts and studies which flow from them will help us much in the intelligent understanding of the causes and development of decay, thereby enabling us the better to prevent it.

*The President.*—Dr. D. D. Smith, of Philadelphia, is present. We shall be glad to hear from him.

*Dr. D. D. Smith.*—I suppose it is my fault that I was not here to hear the paper of the evening, but as I was coming up the street I was disposed to charge my failure to find you to this great city of New York. I found it hard work, indeed, to get on your track, and have been wandering around the streets of your great city consulting policemen and penny-in-the-slot directories until, to my great regret, I have missed the paper entirely. I had fully expected to be here to hear every word of it, because it is a subject in which I feel the greatest interest. I suppose it is hardly fair for me to say anything at all, and cannot attempt a discussion of the paper, for the reason that I know nothing about it; but I know that Dr. Kimball would not have come all the way down from Lake George to hear this paper to-night if he had not the most implicit confidence that the writer of it was thoroughly competent in this department of research; so I assume that the paper reflects really scientific views. And if this be so, of course my views must be in accord with it. Acting upon that presumption, I may be justified in saying a word along the lines upon which Dr. Davenport was speaking when I came in.

New York, with its flaming torch in the outstretched hand of the Goddess of Liberty down in your harbor, is supposed to hold up the beacon of progress not only in commerce, but in science as well. I had expected that everything emanating from New York, even dentistry, was unimpeachable; but I am compelled to take issue with the views which have been presented to-night if they are the accepted views of the profession in New York.

When my paper read before this society in June last is published, there will be found a discussion of this matter of pulpless teeth. I must say I was surprised to hear such stress laid upon this point,—that a tooth with a living pulp is so much superior to what I would call a "pulpless tooth,"—and I ask myself the question, Is there not often a misapprehension as to what the tooth really is? Is it the crown? If the crown is the tooth, it must be admitted that a pulpless tooth is not as good as a tooth with a living pulp. But is the crown the tooth? No. Every one will say the question admits of no argument; the crown is not the tooth. What, then, is the tooth? What is the vital part of the tooth? Take a crown in the most perfect condition and deprive it of its root, or any portion of its root. I do not care whether it has a living pulp or not; has it a root or roots with vital, well-distributed cemental and pericemental issues, and these attached to the alveolar process, and are these cemental and pericemental structures in good condition? If the tooth is not of that character, it matters little what the crown is, *you have not a good tooth.* But suppose you have the root of a tooth in perfect condition, the cementum and pericementum around that root, and the alveolar structure to sustain it, the condition of the pulp or the crown is of trifling account,—you have a good tooth. Where is the man who has practised dentistry who is not able to say that with such a root and such surroundings we can give back to the individual practically as good a tooth as Nature ever put there originally? Is that not so?

I claim that there is not a practitioner in this room who is not demonstrating this fact almost every day of his life. No dentist gives up a tooth because the pulp is dead. Who sends his tooth to the extractor because it has a broken crown? I tell you a large number of teeth are better and more surely saved when without pulps than the same teeth with living pulps. You have never seen nor has the demonstration been made that alveolar pyorrhœa ever began in a pulpless tooth; but you may have seen many teeth which have been rendered perfectly valueless, absolutely lost, through pyorrhœa with perfect living pulps in them. You have seen many of them in which pyorrhœa began and progressed to the destruction and loss of the tooth; but I claim the record is yet to be made of beginning pyorrhœa in a pulpless tooth. Understand what I mean by that. One may have pyorrhœa and afterwards a dead pulp in the tooth, but I say the records are yet to be made of a tooth in good condition as to its alveolar surroundings, and with the pulp destroyed and removed, exhibiting the beginning and progress of pyorrhœa. Hence I say that there are many teeth that are better without pulps than with them. Do not understand that I am advocating the wholesale destruction of pulps to forestall pyorrhœa, and yet I do destroy many pulps by treatment and save many teeth that have been attacked with pyorrhœa, which I believe are unsavable by any other process.

I take the ground, then, gentlemen, that the root of the tooth is the tooth, and that the destruction of the pulp in no way interferes with the cemental structures which give attachment of the tooth to the alveolus; that the cementum and pericementum perform the nutrient function to the root entirely independent of the pulp, and that pulpless roots efficiently treated will be retained in connection with the alveolus indefinitely and in perfect comfort. And is the dental profession ready to admit that, with the multiplicity of engines and instruments, varieties of artificial crowns, and methods for applying them, it is unable to cope with the mechanical problem of affixing a crown to a good root, whether such root be incisor, cuspid, bicuspid, or molar, a crown which shall give back to the mouth the essentials of a tooth in durability, appearance, and utility? I for one am not ready for such admission.

As regards the use of the engine to supplement the stick, there may be on the labial faces of much neglected front teeth accumulations which can be removed with the engine and rubber or felt wheels, but for this periodical treatment the orange-wood stick is much better. The stimulation resulting in improved tooth-structure from this treatment with the stick and pumice is most marked, especially in the mouths of children. The best results are attained by this complete change of environment once in two or four weeks.

*Dr. Bogue.*—I want to express my extreme gratification at the paper that has been read to us this evening. I have had it brought to me more forcibly than ever before (except on one or two occasions) when I tried to tell the truth—tried to tell something I thought I knew, how difficult it is, and now Dr. Hopkins has tried the same thing, and I know he has tried earnestly. He tried to speak the truth every time, yet when he spoke of Dr. Smith's polishing with the stick being so beneficial that the teeth would be aided by it, and would come into their positions, and occupy them in a much better condition than they could in any other way, he omitted a condition that came to my mind, which I want to refer to, and that is a condition of defective formation. Dr. Hopkins will, I am sure, be the first to assent to what I say. I have a skull in my possession, and all the temporary teeth are in it. Most of the permanent teeth are in it also, not all. I was told that the manner of procuring this skull was to have the child shot at the proper age, but of course I do not believe that! Nevertheless, the skull is there. Dr. Davenport has seen it, and also a number of others. The first permanent molars (sixth-year molars) are nearly erupted, and they are in such good condition that we would acknowledge them to be the molars of a civilized child, at least, and to be amenable probably to the treatment suggested in the paper; but when we come to examine the enamel caps of the second molars, which are not yet erupted, and would not have been due for five or six years, as the child lost its life at about six and a half or seven years, we find that they are not perfect by any means. There are crevices between the different layers of enamel. There are sulci, very deep, even to distinct holes. The texture of the enamel is entirely different from that of the first molar, and this difference in formation and structure is perceptible on all the teeth that were in process of formation at the same time. It is a most interesting condition of things to examine, when we undertake to say that a certain process is going to produce and retain immunity from decay. We cannot say that, and we cannot yet express exactly what we mean. I have to the best of my ability made my compliments and thanks and expressed admiration of Dr. Hopkins's way of stating things, and yet he did not explain that. He could not. It is a point well worth considering, however.

*Dr. Davenport.*—I think it would be only fair if our other Boston friend would say a word on the subject.

*Dr. G. F. Eames.*—I am glad for this opportunity to express my admiration for the thoughts that have been given to us to-night by the essayist. I have a profound respect for the man who is able to observe conditions, and then is able, by the use of his chemical analyses and microscope, to demonstrate and come to some satisfactory conclusion by reason of his more technical scientific experiments. I should say, as a result of the paper that has been read to-night, that the essence of the treatment of the condition of dental caries would be embraced under two heads:

1. The promotion to the fullest extent of the function of the teeth as they were designed to do.

2. The bringing about of the proper environment of the teeth by local and general hygienic treatment, which should include the most thorough cleanliness of the teeth and surrounding parts.

I am not prepared to discuss the paper in a general or extensive way. Thank you for the privilege of expressing my admiration of it.

*Dr. D. D. Smith.*—I am very sorry Dr. Bogue felt he must leave at this time. He stated something that I suppose to be in opposition to the spirit of the paper and in opposition to positions which I have taken on this subject of prophylaxis. Therefore, I think it but fair that I should reply to it, although he is not here. I would have been glad to have him remain and hear just what I have to say in regard to the skull. If I may be permitted to express my convictions, I must say the skull with an imperfectly formed undeveloped second molar has no place in the discussion before us. What has imperfect prenatal formation to do with the prevention of decay in the mouth? Nothing, absolutely nothing. What if the enamel, the sulci, and the cusps are imperfect in the preserved skull of a child? What does it show? We must accept conditions not as we would have them, but as they are. It is better to deal with mouths as we find them than to exhaust energy in discussing impossible prenatal betterment.

But dropping this skull, we may properly consider the matter of improving an abnormal articulation in a child. Much can frequently be done in this direction, but you have no control of your patients. Children are not placed unreservedly in the hands of dentists except in special instances. I have had practice enough in dentistry to know that parents do not say to the dentist, "Here, my child is in your hands, do as you think best." I repeat, we must deal with things as they exist in dentistry, whether they relate to articulations, decayed teeth, or prophylaxis. One may talk general principles, and seek to be led by what one conceives to be right, but it will often be found impracticable. The child may come from another operator with tooth extracted, or perhaps has never been to a regular practitioner, but an extractor only; the tooth is out, and you are left to do as you can and not as you would. I can do nothing better for the improvement of articulations than to try and enforce the only true method of keeping the children out of the hands of the extractor.

Take the teeth in any way I have suggested, and change the environment regularly and systematically, hand-polish the teeth on all their surfaces, and put the mouth in a healthy condition, and in nine cases out of ten Nature will do perfect work in keeping the temporary teeth, in the development of the jaw, and in the production of good permanent teeth.

One last word in regard to the devitalization of pulps. Do not understand me as being in favor of destroying pulps in young teeth or in any teeth without an object in view. Pulps in the mouths of young persons especially should be conserved, not destroyed. But we should not lose sight of the fact that when necessity arises and we do destroy the pulp, we do not destroy the teeth. Pulpless teeth can be kept and made good 'way on into old age if we only treat them as they ought to be treated.

*Dr. George S. Allan.*—Very soon, undoubtedly, we will be called upon to pass a vote to Dr. Hopkins for coming here and giving us his most admirable paper on a subject in which all are so deeply interested and all are striving to attain. It strikes out in so many lines and takes in so many points of value and interest, that at this late hour it would be impossible to attempt any discussion of them, or do anything more than express the hope that in some way we may hear from Dr. Hopkins again, if anything further on the subject develops in his mind, and that he will give us some of his latest conclusions, especially on those points as would have reference to our daily practice,—how to attain those results, going more into minutiæ, and bringing out a little more of the points that we would like to work on. It is impossible to touch on all the points in the paper. There is only one which I would briefly allude to, that perhaps the doctor has omitted,—I do not say forgotten, because that is impossible. Take that sublime saying of Pope,—

> "All are but parts of one stupendous whole.
> Whose body Nature is, and God the soul."

We should look upon this little human being as an embodiment of that grand thought, and think of the relationship between the soul and the body, the spirit and substance. When we think how the development of the soul in some mysterious manner depends upon the body, and how the body is by its environment in all possible ways made to suffer and to languish and to fail to fulfil its whole fruition, the thought of longevity is one of prime importance. That being the case, especially in giving man a chance to develop along spiritual lines, there is nothing impresses me more as I grow older in my work and see more of it than the absolute necessity, if man is to keep to his full length of years, than the preservation of the teeth in a working manner. There is nothing on which the stomach so much depends for nutriment in its proper shape to strengthen the body, as that the teeth should properly prepare the food, and lives are shortened by the absence of these most necessary organs to prepare the food, so that middle age and old age, even extreme old age, will be made useful not only to the individual, but to all mankind. I would like to have the privilege of offering Dr. Hopkins our sincere thanks for presenting this paper to us in this most masterly manner, in a way in which we can all understand it.

A vote of thanks was tendered to Dr. Hopkins.

*The President.*—Does Dr. Hopkins wish to say anything in closing the discussion?

*Dr. Hopkins.*—There is not much more to say. One gentleman has answered another and cleared up most of the points. This peculiar ropy saliva I have at times thought was connected with catarrhal trouble, and at other times stomach trouble. It seems to me that anything that causes irritation of the mucous membrane is apt to cause an increase in the thickness of the saliva. There are a number of bacteria, not especially acid-producing forms, frequently found in the mouth that would thicken the saliva, and make it more viscid and ropy, and it has seemed to me that this ropiness might come from bacterial activity,—not, however, from the activity of acid-producing bacteria. I have not cleared it up in my own mind, but there is one thing that has been evident to me, that whatever may be the cause of this ropiness, you will usually find that the gelatin plaques that Williams and Black, as I think, lay undue stress upon, and which are associated with caries, are found more frequently in mouths that have this viscid, ropy saliva, and whatever causes this condition of viscidity causes also the gelatin plaques. But that I do not think is acid-producing bacteria. I think that what Dr. Bogue said in regard to the enamel of the second molar in the case he mentioned can be seen very much earlier in life. In fact, as far back as embryonic life, the differences in the structure of the teeth are quite well marked, so that you might prognosticate the kind of teeth a child would have were it possible to examine them before they develop; particularly is this true where there is an interruption of nutrition. It may happen in prenatal life that some illness affecting the mother may cause an interruption in nutrition. This will cause an apparent line of demarcation in the enamel, and will leave it in an impaired condition when the tooth erupts. Next time I have a paper to read on this subject, Dr. Smith and I will have to get together outside, so as to be sure we agree as to what we are going to impress upon the society. I think we do agree in almost everything. Certainly the differences in details are so slight that they need not affect the importance of our work. Our object, I am sure, is mainly to create better ideals. Every man can work the idea out for himself if he has the force from behind that will induce him to take hold of the matter. It will probably be found that individual methods will suit individuals better, and that no one broad scheme can be laid down that every man can bind himself to. One must study the conditions as they arise, but the important thing is to keep our ideals so high that we will have to keep climbing all the time to reach them, and in that way, I think we will accomplish what we are aiming at.

*Dr. Kimball.*—A suggestion has been made, and I would like to express it as the hope of the society, that in the publication of this paper Dr. Hopkins can arrange so that his three papers may be printed together. I know some of us would be very glad to have such reprints.

Adjourned.

FRED. L. BOGUE, M.D., D.D.S.,
*Editor The New York Institute of Stomatology.*

